# Predictors of failure to rescue in surgical rescue

**DOI:** 10.1007/s00423-026-03986-8

**Published:** 2026-02-23

**Authors:** Katsuhiro Ogawa, Yuji Miyamoto, Yuki Hisano, Yuto Maeda, Mayuko Ohuchi, Yukiharu Hiyoshi, Satoshi Ida, Masaaki Iwatsuki

**Affiliations:** Department of Gastroenterological Surgery, Graduate School of Life Science, Kumamoto University, 1-1-1 Honjo, Chuo-ku, Kumamoto, 860-8556 Japan

**Keywords:** Surgical rescue, Failure to rescue, Surgical rescue severity score, Acute care surgeon, Acute care surgery

## Abstract

**Aim:**

To determine the current status of surgical rescue (SR) and examine the predictors of failure to rescue (FTR). In acute care surgery, predictors of FTR have only been reported in patients with trauma, not in SR.

**Methods:**

This retrospective study included 142 patients who underwent emergency surgery at our institution between April 2019 and March 2023. The primary outcome was the identification of the FTR predictors using logistic regression analysis. The secondary outcome was the development of a Surgical Rescue Severity Score (SRSS) using these SR predictors and long-term outcomes.

**Results:**

The patients were divided into survival and mortality groups (122 and 20, respectively). American Society of Anesthesiologists Physical Status (ASA-PS) and quick sequential organ failure assessment (qSOFA) scores were higher in the mortality group. In contrast, the survival group included more cases of SR necessitated by surgical treatment. In multivariate analysis, ASA-PS ≥ 3 (odds ratio [OR], 5.3; 95% confidence interval [CI] 1.2–36), complications caused by non-surgical therapy (OR, 5.5; 95% CI 1.8–19), and qSOFA score ≥ 2 (OR, 4.5; 95% CI 1.3–17) were independent prognostic factors. The total risk factors for FTR obtained in the multivariate analysis were calculated and established as the SRSS. The long-term prognosis of SR was examined and stratified by the SRSS (*P* = 0.0001).

**Conclusion:**

Predictors of FTR in SR were ASA-PS ≥ 3, qSOFA score ≥ 2, and complications caused by non-surgical therapy. The SRSS stratified the short- and long-term prognoses of SR and predicted prognoses preoperatively.

## Introduction

Acute Care Surgery (ACS) was first proposed by The American Association for the Surgery of Trauma in 2005 as a new surgical field with trauma surgery, emergency general surgery, and critical surgical care as its three pillars [1–3]. More recently, surgical rescue (SR), which involves intensive management and surgical treatment of patients with postoperative complications, has gained recognition as the fourth pillar [4, 5]. 

SR involves rescue procedures to address complications after an invasive procedure or surgery [4]. Patients requiring SR are referred not only from their own departments but also from other departments in the same facility and other facilities [4]. Furthermore, patients requiring SR have been reported to have longer lengths of stay and higher in-hospital mortality rates than patients in other ACS departments. However, SR is a new concept; its definition is vague, and few studies have evaluated it.

The concept of failure to rescue (FTR), defined as the number of deaths among patients with complications, was first reported by Silber et al. in 1992 [6]. In the field of ACS, FTR has been reported in patients with trauma [7] but not in those with SR. Therefore, in this study, we aimed to determine the current status of SR, examine the predictors of FTR in cases of SR, and develop a scoring system for SR.

## Methods

### Participants

We enrolled 142 patients who underwent general emergency surgery for SR at the Department of Gastroenterological Surgery at Kumamoto University Hospital between April 2019 and March 2023. The patients were divided into an in-hospital death group (mortality group) and a live-discharge group (survival group), and the findings for the two groups were compared. This study was approved by the Human Ethics Review Committee of the Kumamoto University Hospital (approval no. 2775). SR is generally defined as a rescue procedure for complications following invasive procedures or surgeries [4]. In this study, SR was considered surgical salvage for complications of surgery or invasive procedures (endoscopy, interventional radiology [IVR], catheter placement, radiation therapy, and chemotherapy). FTR was defined as the in-hospital death of a patient after SR.

## Data collection

We collected baseline data for all patients, including information related to the patient’s age, sex, body mass index (BMI), American Society of Anesthesiologists Physical Status (ASA-PS), complication origin, cause and treatment of the complications, involvement of acute care surgeons, and door-to-incision time (DTIT). In addition, the patient’s white blood cell (WBC) counts, platelet (Plt) counts, and C-reactive protein (CRP), creatinine (Crea), and total bilirubin (T-bil) levels were determined using blood tests. Furthermore, details of preoperative scores (quick sequential organ failure assessment [qSOFA] and sequential organ failure assessment [SOFA] scores) and surgery-related factors (operation time, blood loss, laparoscopic surgery, and damage control surgery/open abdominal management [DCS/OAM]) were recorded.

In this study, the term “acute care surgeon” was used to describe the following groups of medical professionals: (1) board-certified surgeons registered with the Japan Surgical Society and (2) acute care surgery-certified surgeons registered with the Japanese Society for Acute Care Surgery.

## Outcomes

The primary outcome of this study was to identify predictors of FTR in patients undergoing SR, using multivariate logistic regression analysis. The secondary outcome was to develop a novel scoring system—the Surgical Rescue Severity Score (SRSS)—based on the identified FTR predictors, as well as short-term and long-term patient outcomes following SR. Long-term prognosis was defined as 3-year survival. Additionally, subgroup analyses were performed based on the etiology of SR (surgery-related vs. non-surgical complications) and on whether an acute care surgeon was involved in the case.

## Development of the surgical rescue severity score (SRSS)

To our knowledge, no existing scoring system has been validated specifically for predicting FTR in the context of surgical rescue. Therefore, we developed the Surgical Rescue Severity Score (SRSS) as an original, institution-based scoring model derived from the results of multivariate logistic regression analysis.

Three independent prognostic factors for in-hospital mortality were identified and incorporated into the SRSS:

1. ASA-PS classification ≥ 3.
2. qSOFA score ≥ 2.
3. Complications caused by non-surgical therapy (e.g., endoscopy, IVR, catheter).

One point was assigned for the presence of each factor, yielding a total SRSS ranging from 0 to 3. A higher SRSS corresponded with a higher observed in-hospital mortality rate in our cohort. The score is based on commonly used clinical indicators and is therefore simple, intuitive, and reproducible across diverse acute care settings. The discriminative performance of the SRSS was evaluated using a receiver operating characteristic (ROC) curve, and the area under the curve (AUC) was calculated.

## Statistical analyses

Variables are presented as median (25–75% interquartile range) or percentage of patients. Univariate analyses were performed using the chi-squared test for categorical variables and the Mann–Whitney U-test for continuous variables. In all tests, the significance level for two-tailed P-values was set at *P* < 0.05. We used JMP version 10.0.2 (SAS Institute, Cary, NC, USA) for the statistical analyses. A multivariate analysis of in-hospital mortality was performed using logistic regression. The cut-off values for age, ASA-PS, SOFA score, Crea level, and blood loss were determined using median values. The cut-off value for the qSOFA score was 2, based on the Sepsis-3 definition [8]. The factors included in the multivariate analysis were age, sex, ASA-PS, complications caused by non-surgical therapy, involvement of an acute care surgeon, qSOFA score, SOFA score, Crea level, and blood loss. Multivariate logistic regression analysis with backward elimination was performed for variables with a significance of *P* < 0.10 in the univariate analysis to calculate odds ratios (ORs) and 95% confidence intervals (CIs). The statistical significance was set to *P* < 0.05. All variables were fully recorded, and there were no missing data in the dataset.

## Results

### Clinical patient characteristics

Table 1 presents the patient background characteristics. The data of 142 patients were analyzed during the study period. The median patient age was 69 years, and 61% of the patients were men. Surgery was the most common cause of SR (82 patients, 58%), followed by chemotherapy (30 patients, 21%). In addition, SR was necessitated by IVR in nine patients, endoscopy in eight, and radiotherapy in six. Complications originated from our department in 66 patients (46%), from other departments in 67 (47%), and other hospitals in nine (6%). Peritonitis was the most common complication (52 patients, 37%), followed by bowel obstruction (30 patients, 21%) and bleeding (21 patients, 15%). Regarding surgical details, small bowel resection, colorectal resection, and drainage were performed in 24 patients (17%), colorectal resection in 20 (14%), drainage in 19 (13%), bowel adhesiolysis in 17 (12%), and cholecystectomy and hemorrhage control in 14 (10%).

**Table 1:** Patients’ characteristics

Age (years old), median (IQR)	69 (60–74)
Male, *n* (%)	87 (61)
BMI (kg/m2), median (IQR)	21 (19–24)
ASA-PS	3 (2–3)
Complication origin	
Our department	66 (46)
Other departments	67 (47)
Other hospitals	9 (6)
Cause treatment	
Surgery	82 (58)
Chemotherapy	31 (22)
IVR	9 (6)
Endoscopy	8 (6)
Radiation	6 (4)
Others	6 (4)
Complications	
Peritonitis	52 (37)
Bowel obstruction	30 (21)
Bleeding	21 (15)
Mesenteric ischemia	18 (13)
Cholecystitis	14 (10)
Appendicitis	2 (1)
Others	5 (4)
Treatment	
Small bowel resection	24 (17)
Colorectal resection	20 (14)
Drainage	19 (13)
Adhesiolysis	17 (12)
Cholecystectomy	14 (10)
Hemorrhage control	14 (10)
Stoma creation	13 (9)
Omental pack	9 (6)
Others	12 (8)

*IQR* interquartile range, *BMI* body mass index, *ASA-PS* american society of anesthesiologists physical status, *IVR* interventional radiology.

Table 2 shows a comparison of the patient background characteristics between the survival (*n* = 122) and mortality (*n* = 20) groups. Age, sex, and BMI did not differ significantly between the two groups. The ASA-PS was significantly higher in the mortality group than in the survival group (3 [2]– [3] vs. 3 [3]– [4]). The two groups showed no significant differences in the presence or absence of malignant disease, the origin of complications, or details of the complications. When cases were categorized by the cause of SR, the survival group included significantly more cases of complications associated with surgery than the death group. The two groups showed no significant differences in acute care surgeon involvement or DTIT scores. The groups also showed no significant differences in preoperative WBC and Plt counts as well as CRP and T-bil levels; however, Crea levels were significantly higher in the mortality group. The preoperative qSOFA and SOFA scores were significantly higher in the mortality group. Among surgery-related factors, operative time, blood loss, percentage of laparoscopic procedures, or percentage of DCS/OAM did not differ significantly between the groups.

**Table 2:** Comparison of patients’ characteristics between the two groups

	Survival group (*n* = 122)	Mortality group (*n* = 20)	*p* value
Age (years old), median (IQR)	69 (60–74)	70 (62–74)	0.73
Male, *n* (%)	75 (61)	12 (60)	1.00
BMI (kg/m^2^), median (IQR)	21 (19–24)	21 (18–24)	0.65
ASA-PS	3 (2–3)	3 (3–4)	< 0.001
Complication origin			0.71
Our department	55 (45)	11 (55)	
Other departments	59 (48)	8 (40)	
Other hospitals	8 (7)	1 (5)	
Complications			0.22
Peritonitis	42 (34)	10 (50)	
Bowel obstruction	29 (24)	1 (5)	
Bleeding	17 (14)	4 (20)	
Mesenteric ischemia	14 (11)	4 (20)	
Cholecystitis	14 (11)	0 (0)	
Appendicitis	2 (2)	0 (0)	
Other	4 (3)	1 (5)	
Cause treatment			0.03
Surgery	77 (63)	5 (25)	
Chemotherapy	24 (20)	7 (35)	
Endoscopy	6 (5)	2 (10)	
IVR	7 (6)	2 (10)	
Radiation	4 (3)	2 (10)	
Others	4 (3)	2 (10)	
Involvement of acute care surgeon	36 (30)	7 (35)	0.60
DTIT (min), median (IQR)	286 (62–490)	284 (193–498)	0.58
《Preoperative labo data》			
WBC(/µl), median (IQR)	10,050 (6275–13825)	8050 (4600–15675)	0.35
Plt(×10^4^/L), median (IQR)	6 (1–14)	8 (4–18)	0.13
CRP(mg/dL), median (IQR)	20 (15–29)	18 (7–25)	0.19
Crea(mg/dL), median (IQR)	0.8 (0.6–1.1)	1.2 (0.8–2.1)	0.04
T-bil(mg/dL), median (IQR)	0.8 (0.5–1.2)	0.9 (0.6–1.6)	0.29
《Preoperative scoring》			
qSOFA score, median (IQR)	1 (0–1)	2 (0–3)	0.003
SOFA score, median (IQR)	1 (0–3)	3 (1–8)	0.002
《Surgery related factor》			
Operation time (min), median (IQR)	132 (96–170)	117 (90–221)	0.92
Blood loss (mL), median (IQR)	49 (10–251)	97 (6–1533)	0.29
Laparoscopic surgery, n (%)	40 (33)	3 (15)	0.12
DCS/OAM	5 (4)	0 (0)	1.00

*IQR* interquartile range, *BMI* body mass index, *ASA-PS* american society of anesthesiologists physical status, *IVR* interventional radiology, *DTIT* door to incisional time, *WBC* white blood cell, *Plt* platelet, *CRP* C-reactive protein, *Crea* creatinine, *T-bil* total bilirubin, *SOFA* sequential organ failure assessment, *DCS* damage control surgery, *OAM* open abdominal management.

## Primary outcomes

The primary outcomes are presented in Table 3. Logistic regression analysis was used to examine the risk factors for in-hospital mortality. In the univariate analysis, ASA-PS ≥ 3, complications caused by non-surgical therapy, qSOFA score ≥ 2, SOFA score ≥ 2, and Crea level ≥ 0.8 were identified as risk factors. In the multivariate analysis, ASA-PS ≥ 3 (OR, 5.3; 95% CI, 1.2–36), complications caused by non-surgical therapy (OR, 5.5; 95% CI, 1.8–19), and qSOFA score ≥ 2 (OR, 4.5; 95% CI, 1.3–17) were independent prognostic factors.

**Table 3:** Logistic regression analysis for in-hospital mortality

	Univariate analysis			Multivariate analysis		
	OR	95% CI	*P* value	OR	95% CI	*P* value
Age ≥ 70 years old	1.5	0.57–3.94	0.41			
Male	0.94	0.36–2.55	0.9			
ASA-PS ≥ 3	7.9	2.2–51	< 0.001	5.3	1.2–36	0.03
Caused by non-surgical therapy	5.1	1.85–16.6	0.001	5.5	1.8–19	0.003
Involvement of acute care surgeon	1.3	0.45–3.4	0.62			
qSOFA ≥ 2	5.4	1.9–15	0.001	4.5	1.3–17	0.02
SOFA score ≥ 2	3	1.1–9.1	0.03			
Crea ≥ 0.8 mg/dL	3	1.1–9.7	0.03			
Blood loss ≥ 50mL	1.5	0.57–4.1	0.4			

*CI* Confidence interval, *SOFA* sequential Organ Failure Assessment, *ER* emergency room.

### Secondary outcome

**Fig. 1:**
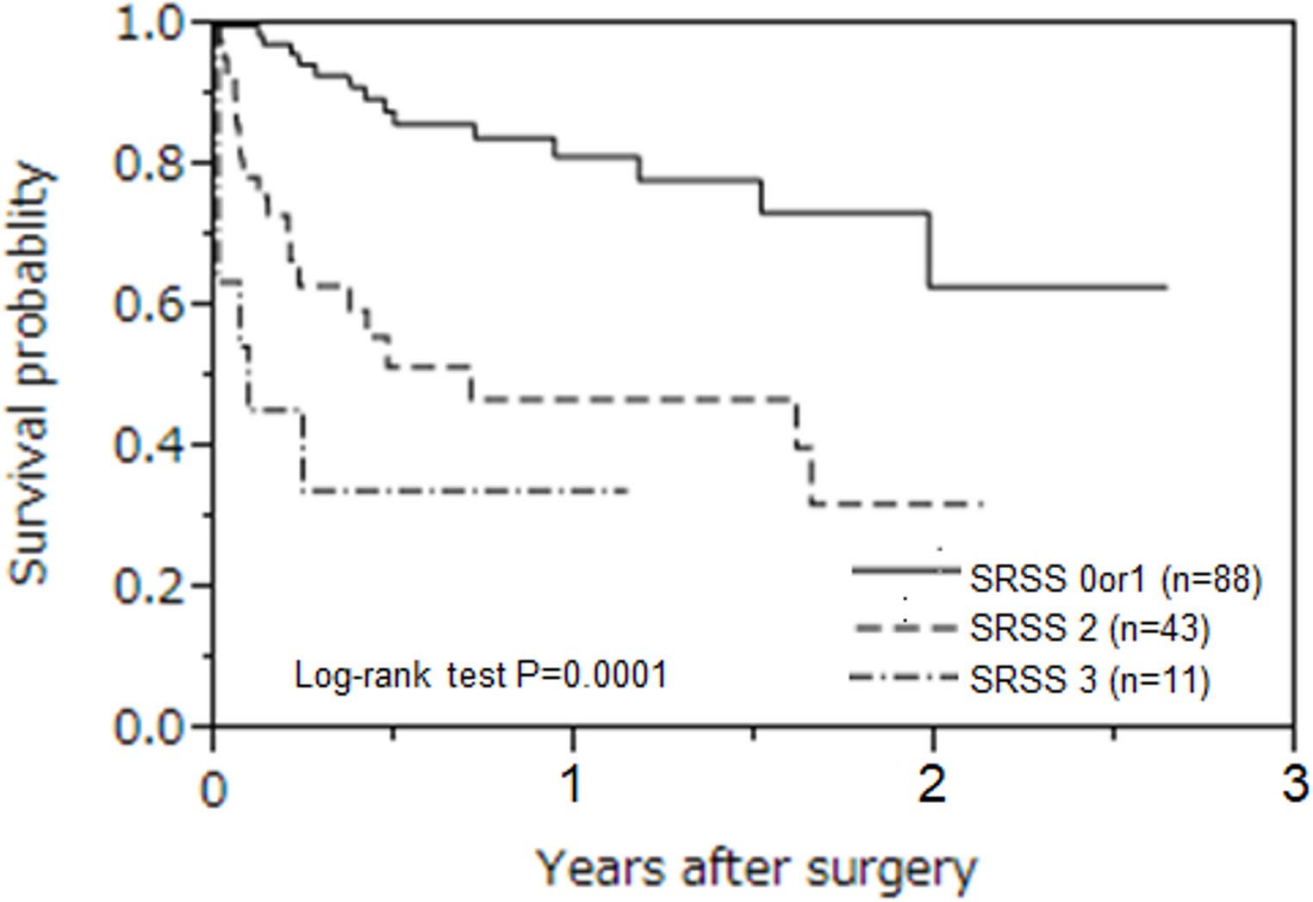
Long-term prognosis of surgical rescue The long-term prognosis of SR was examined and stratified by the SRSS (log-rank test, *P* = 0.0001)

**Fig. 2:**
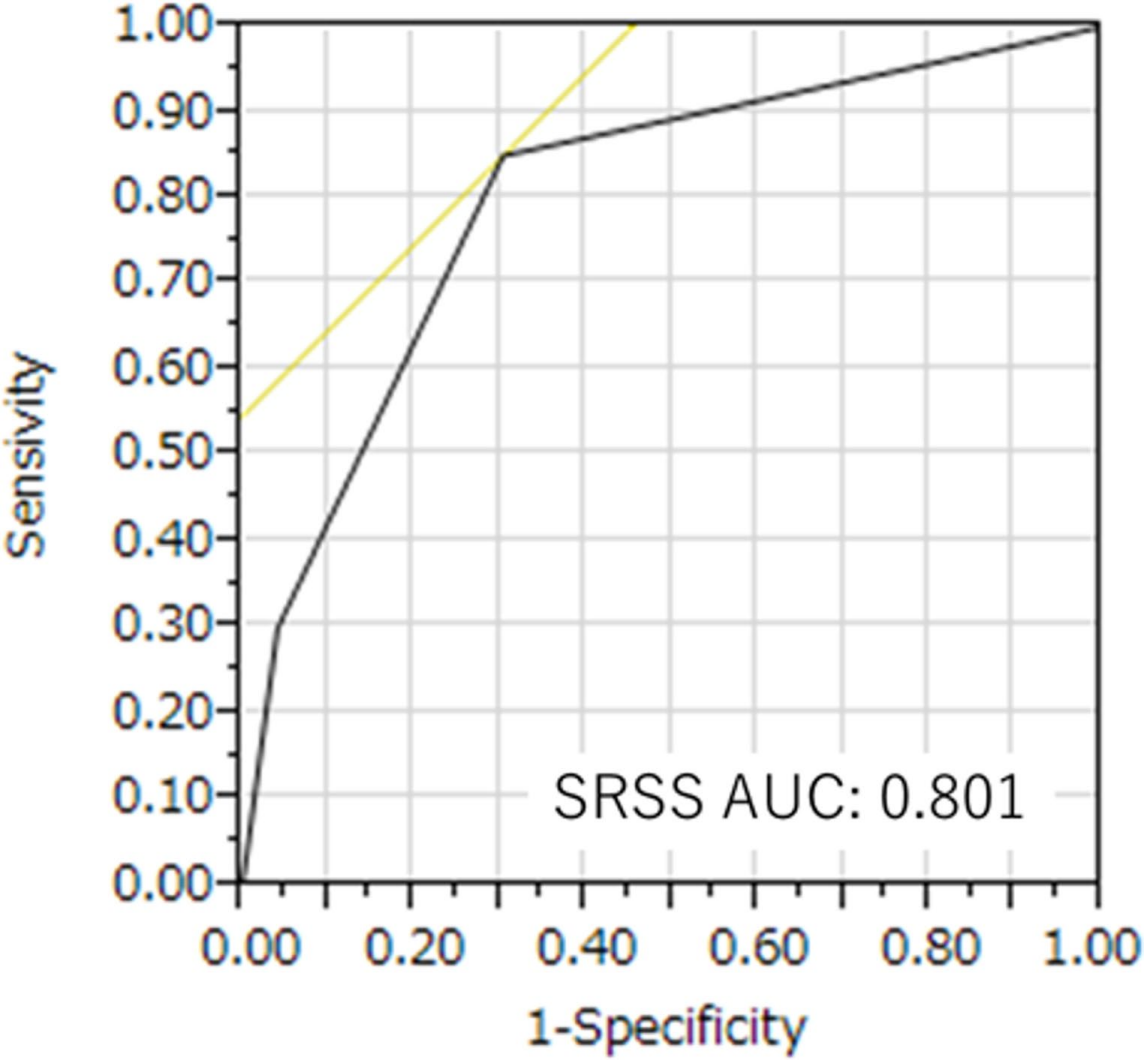
ROC Curve and AUC for SRSS in Predicting In-Hospital Mortality The ROC curve demonstrates the discriminatory performance of the Surgical Rescue Severity Score (SRSS) for predicting in-hospital mortality. The AUC was 0.801, indicating good predictive accuracy

The secondary outcomes are presented in Table 4. The total risk factors for in-hospital mortality obtained in the multivariate analysis were calculated and established as a new scoring system. We named this the SRSS. To calculate in-hospital mortality, SRSS values of 0 and 1 were stratified as 3%, an SRSS value of 2 was stratified as 26%, and an SRSS value of 3 was stratified as 55%. The ORs were 10 (95% CI, 2.8–45) for an SRSS of 2 and 34 (95% CI, 7–206) for an SRSS of 3. The long-term prognosis of SR was also examined and stratified by SRSS (Fig. 1, log-rank test, *P* = 0.0001). To more precisely assess the discriminatory ability of the SRSS in predicting in-hospital mortality, we supplemented the analysis with a ROC curve and calculated the AUC, as presented in Fig. 2.

**Table 4:** Logistic regression analysis of SRSS for in-hospital mortality

Univariate analysis				In-hospital mortality (%)
SRSS	OR	95% CI	*P* value	
0 or 1	1			3/88 (3)
2	10	2.8–45	< 0.001	11/43 (26)
3	34	7.01–206	< 0.001	6/11 (55)

*SRSS* surgical rescue severity score, *OR* odds ratio, CI,.

### Subgroup analysis

Patient background characteristics were compared based on whether the cause of SR was surgical or non-surgical therapy (Table 5). The two groups showed no differences in age, sex, or ASA-PS; however, the non-surgery group had a significantly lower BMI. The non-surgery group involved other departments and hospitals as the origin of complications. The non-surgery group also showed a higher incidence of peritonitis. Acute care surgeon involvement and DTIT scores were not significantly different between the groups. Preoperative laboratory data showed that the non-surgical group had significantly lower WBC and Plt counts. The qSOFA and SOFA scores did not significantly differ between the groups. The two groups also showed no significant differences in surgery-related factors.

**Table 5:** Comparison of patients’ characteristics between the surgery and non-surgery groups

	Surgery group (*n* = 82)	Non-surgery group (*n* = 60)	*p* value
Age (years old), median (IQR)	70 (60–76)	67 (61–73)	0.23
Male, *n* (%)	55 (67)	32 (53)	0.11
BMI (kg/m^2^), median (IQR)	22 (19–25)	20 (18–23)	0.02
ASA-PS	3 (2–3)	3 (2–3)	0.26
Complication origin			0.06
Our department	45 (55)	21 (35)	
Other departments	33 (40)	34 (57)	
Other hospitals	4 (5)	5 (8)	
Peritonitis	25 (30)	28 (47)	0.06
Involvement of acute care surgeon	23 (28)	20 (33)	0.58
DTIT (min), median (IQR)	262 (151–437)	307 (168–533)	0.18
《Preoperative labo data》			
WBC(/µl), median (IQR)	11,300 (7500–13925)	7000 (4925–14225)	0.02
Plt(×10^4^/L), median (IQR)	21.7 (16.6–31.5)	18.4 (9.4–24.6)	0.008
CRP(mg/dL), median (IQR)	6.7 (1.7–17)	5.4 (0.7–13)	0.34
Crea(mg/dL), median (IQR)	0.8 (0.6–1.2)	0.9 (0.6–1.3)	0.38
T-bil(mg/dL), median (IQR)	0.9 (0.5–1.3)	0.7 (0.6–1.4)	0.39
《Preoperative scoring》			
qSOFA score, median (IQR)	1 (0–1)	1 (0–1)	0.80
SOFA score, median (IQR)	1 (0–3)	2 (0–5)	0.16
《Surgery related factor》			
Operation time (min), median (IQR)	133 (96–170)	129 (94–186)	0.79
Blood loss (mL), median (IQR)	58 (11–280)	42 (7–300)	0.48
Laparoscopic surgery, n (%)	24 (29)	18 (30)	1.00

*IQR *interquartile range, *BMI* body mass index, *ASA-PS*, american society of anesthesiologists physical status, *IVR* interventional radiology, *DTIT* door to incisional time, *WBC* white blood cell, *Plt* platelet, *CRP* C-reactive protein, *Crea* creatinine, *T-bil* total bilirubin, *SOFA* sequential organ failure assessment.

Patient background characteristics in cases with and without the involvement of an acute care surgeon were also compared separately (Table 6). The two groups showed no significant differences in age, BMI, or ASA-PS; however, the acute care surgeon group included significantly more women. In addition, the acute care surgeon group included more referrals from other departments and hospitals. The two groups showed no differences in complications from non-operative therapy, peritonitis, or DTIT. They also showed no differences in the preoperative laboratory data, but the qSOFA score was significantly higher in the acute care surgeon group. The incidence of SRSS scores of 2 or 3 was higher, but the difference was not significant. The two groups showed no differences in surgery-related factors.

**Table 6:** Comparison of patients’ characteristics between the acute care surgeon and non-acute care surgeon groups

	Acute care surgeon group (*n* = 43)	Non-Acute care surgeon group (*n* = 99)	*p* value
Age (years old), median (IQR)	69 (60–76)	69 (62–73)	0.72
Male, *n* (%)	17 (40)	70 (71)	< 0.001
BMI (kg/m^2^), median (IQR)	21 (19–24)	21 (19–24)	0.59
ASA-PS ≥ 3, n (%)	24 (56)	59 (60)	0.71
Complication origin: our department	15 (35)	51 (52)	0.09
Caused by non-surgical therapy	20 (47)	40 (40)	0.58
Peritonitis	19 (44)	34 (34)	0.34
DTIT (min), median (IQR)	268 (162–442)	293 (163–495)	0.77
《Preoperative labo data》			
WBC(/µl), median (IQR)	7900 (5700–16000)	10,400 (6200–13600)	0.73
Plt(×10^4^/L), median (IQR)	20 (10–26)	20 (15–30)	0.35
CRP(mg/dL), median (IQR)	4.8 (0.7–18)	6.8 (2.1–14)	0.64
Crea(mg/dL), median (IQR)	0.85 (0.51–1.21)	0.81 (0.61–1.21)	0.68
T-bil(mg/dL), median (IQR)	0.7 (0.6–1.2)	0.9 (0.5–1.3)	0.53
《Preoperative scoring》			
qSOFA score, median (IQR)	1 (0–2)	0 (0–1)	0.01
qSOFA ≥ 2, n (%)	13 (30)	16 (16)	0.07
SOFA score, median (IQR)	2 (0–6)	1 (0–3)	0.21
SRSS, medial (IQR)	1 (0–2)	1 (1–2)	0.34
SRSS 2/3, n (%)	20 (47)	34 (34)	0.19
《Surgery related factor》			
Operation time (min), median (IQR)	130 (97–160)	130 (91–177)	0.85
Blood loss (mL), median (IQR)	75 (20–300)	40 (10–280)	0.19
Laparoscopic surgery, n (%)	16 (37)	27 (27)	0.24

*IQR* interquartile range, *BMI* body mass index, *ASA-PS* american society of anesthesiologists physical status, *DTIT* door to incisional time, *WBC* white blood cell, *Plt* platelet, *CRP* C-reactive protein, *Crea* creatinine, *T-bil* total bilirubin, *qSOFA* quick sequential organ failure assessment, *SOFA* sequential organ failure assessment, *SRSS* surgical rescue severity score.

## Discussion

In the present study, ASA-PS ≥ 3, qSOFA score ≥ 2, and complications caused by non-surgical therapy were identified as predictors of FTR in SR. In addition, our SRSS stratified the short- and long-term prognoses of SR, suggesting that it may be useful for preoperative risk assessment.

Only a few epidemiological studies on SR have been published to date. Briggs et al. reported that cases of SR accounted for 13% of the inpatient cases treated by an ACS team [9]. In their study, 85% of the SR cases showed complications from surgery, while the remaining 15% showed complications from procedures or endoscopy. In our study, 82 (58%) cases showed surgery-related complications. Kutcher et al. reported that SR was required in 320 (13%) of 2,410 patients who underwent ACS [4]. Among the cases involving SRs, 36% were referred by their own departments, 38% by other departments, and 26% by other hospitals. Moreover, compared with patients in other ACS departments, those requiring SR had longer hospital stays and higher in-hospital mortality rates. However, the definition of SR remains ambiguous. Although SR is considered a remedy for invasive procedures and postoperative complications [4], the details of invasive procedures have not yet been defined. In this study, we included chemotherapy, radiotherapy, and IVR procedures as invasive procedures. Thus, establishing a clear definition of SR is essential.

Ghaferi et al. compared mortality outcomes between hospitals after the following six major surgeries: pancreatectomy, esophagectomy, abdominal aortic aneurysm surgery, coronary artery bypass surgery, aortic valve replacement, and mitral valve replacement [10]. They concluded that complication rates were equal among hospitals, but mortality rates after complications differed and that better management of complications contributed to lower mortality rates. Peitzman et al. reported that more than 80% of patients with SR required surgery, and 50% required multiple surgeries [5]. Moreover, over half of these patients were admitted to the intensive care unit. They reported that a team of experienced surgeons is required to handle many of these complex complications and critical illnesses and that an acute care surgeon should be in charge. Khalil et al. reported that trauma centers staffed by acute care surgeons were more effective in reducing the length of stay, medical costs, and complications in emergency general surgery than regular trauma centers [11]. In our study, the involvement of acute care surgeons in SR did not improve the outcomes. This finding can be attributed to the following reasons: First, acute care surgeons might have treated patients with severe SR. The acute care surgeon group had more referrals from other departments and hospitals, and the qSOFA score in this group was significantly higher. An SRSS score of 2 or 3 was also more common in the acute care surgeon group. In a previous study, Kevin et al. reported that surgeons with more years of experience were entrusted with emergency surgeries for more severely ill patients, such as those with septic shock and renal failure [12]. Second, only one acute care surgeon was involved in the study. The number of acute care surgeons in Japan is small, and increasing the number of such surgeons is important to reduce the rates of FTR in SR.

The ASA-PS [13–15] and qSOFA score [16–18], which were identified as predictors of FTR in SR in this study, have been reported as prognostic factors for other diseases. Sato et al. tested whether the ASA-PS could predict the prognosis of 301 patients with esophageal cancer who underwent esophagectomy [15]. In their study, multivariate analysis identified the ASA-PS as an independent predictor of overall survival (OS) in patients with esophageal cancer. Patients with an ASA-PS of 3 reportedly showed a lower rate of perioperative chemotherapy, which was attributed to longer operative times and greater blood loss in these patients. The authors concluded that more careful perioperative management is needed in patients with an ASA-PS of 3. Endo et al. reported the findings for patients with gastric cancer aged > 80 years who underwent gastrectomy [14]. In their study, an ASA-PS of 3/4 was reported to be an independent predictor of OS. Both reports cited the ASA-PS as a predictor of long-term prognosis after elective surgery. Our study is the first to identify ASA-PS as a predictor of the short-term prognosis of SR, one of the pillars of ACS. Abdullah et al. reported the findings for 434 patients with sepsis diagnosed using the Systemic Inflammatory Response Syndrome criteria [18]. They reported that a qSOFA score ≥ 2 was an independent prognostic predictor of 30-day mortality. The qSOFA score was proposed as a diagnostic criterion for sepsis in the emergency room in Sepsis-3 [8]. Previous studies have generally reported that the qSOFA score has high specificity and low sensitivity for predicting mortality in patients with sepsis [19]. In this study, we report the importance of the qSOFA score as a prognostic factor for SR. Previous studies have reported the usefulness of the qSOFA score as a prognostic factor in diseases other than sepsis, such as acute pulmonary embolism [20] and idiopathic pulmonary fibrosis [21]. This is the first study to report the utility of the qSOFA score in SR.

Complications caused by non-operative therapy were also identified as predictors of FTR in this study. The results indicated that cases of SR necessitated by non-operative therapy were referred from other departments or hospitals. Kutcher et al. reported that patients from other departments had similar in-hospital mortality and long-term outcomes but worse length of stay and home discharge rates than patients from their department [4]. The complications caused by non-operative therapy might have been a prognostic factor in the present study as well because many patients were referred from other departments and hospitals. The non-surgery group showed a lower BMI, more peritonitis, and lower WBC and Plt counts. This suggests that the non-surgery group might have been in a worse condition than the surgery group.

Only one study of FTR in ACS has been previously reported. Abe et al. reported the complications and in-hospital mortality rates in patients with trauma [7]. The Japan Trauma Data Bank was used in their study, and 184,214 trauma cases were included. They reported lower complication and FTR rates in high-volume trauma centers. Additionally, they emphasized that avoiding trauma deaths, as well as complications, is important to improve the quality of trauma care. This study is the first to report FTR in cases involving SR. The predictors of FTR in SR were qSOFA score ≥ 2, ASA-PS ≥ 3, and complications caused by non-surgical therapy. Moreover, the SRSS based on these predictors can predict the prognosis from the preoperative stage. In this study, the in-hospital mortality rate for patients with an SRSS score of 0 was 0%, and for a score of 1 it was 3%, both representing very low-risk groups. In contrast, mortality increased markedly with higher scores—26% for score 2 and 54% for score 3—demonstrating a clear risk gradient. Notably, due to the absence of in-hospital deaths in the score 0 group, the odds ratio (OR) could not be calculated. To address this limitation and enhance model stability, we combined the 0- and 1-point groups, both of which exhibited similarly low mortality rates. The area under the ROC curve for SRSS was 0.801, indicating strong discriminatory power and supporting its utility as a prognostic tool for predicting in-hospital mortality.

This study had some limitations. First, it was a retrospective, single-center analysis conducted at a small-scale facility, with a total sample size of 142 patients and only 20 failure-to-rescue (FTR) events. Although multivariate analysis identified significant predictors of FTR, the wide CIs of some odds ratios (e.g., ASA-PS: 1.2–36.0) indicate imprecision in the estimates. These broad CIs reflect the limited statistical power of the study and raise concerns regarding the stability and generalizability of the results. Therefore, while the identified associations provide valuable insights, they should be interpreted with caution. Future research involving larger, multicenter cohorts is necessary to validate the present findings and enhance the external validity and predictive accuracy of the proposed model. Second, the definition of SR remains ambiguous. The invasive procedures discussed in this study included chemotherapy, radiation therapy, and IVR procedures, as mentioned earlier. In the few reports that have been published in the past, IVR was often included in invasive procedures, but chemotherapy and radiation therapy were not. However, in real practice, especially in the field of oncologic emergencies, treatment of complications after chemotherapy and radiotherapy is common and is expected to increase in the future due to its expanding indications. Therefore, acute care surgeons need to be familiar with these diseases, which is why they were included in the SR in this study. Future studies should aim to develop a clear definition of SR. Third, only one acute care surgeon was involved; therefore, the usefulness of acute care surgeons in SR could not be demonstrated. Although a few reports have described improved prognoses with the involvement of acute care surgeons in SR, this issue needs to be explored in more detail in future studies. Fourth, because the surgical rescue cases were extracted from the gastroenterological surgery registry, patients who were eligible for surgical rescue but were not rescued were not included. Selection bias may be at play here.

The predictors of FTR in SR were ASA-PS ≥ 3, qSOFA score ≥ 2, and complications caused by non-surgical therapy. The SRSS developed in this study can stratify the short- and long-term prognoses of SR and predict the prognosis even before surgery.

## Data Availability

The data underlying this article will be shared by the corresponding author upon reasonable request.
